# A comprehensive overview of neuropsychiatric symptoms in adolescents with 22q11.2 deletion syndrome

**DOI:** 10.1111/jir.13196

**Published:** 2024-10-22

**Authors:** I. Selten, J. Blok, T. Boerma, A. A. A. M. J. Djelantik, M. Houben, F. Wijnen, J. Zinkstok, J. A. S. Vorstman, A. M. Fiksinski

**Affiliations:** ^1^ Institute for Language Sciences Utrecht University Utrecht The Netherlands; ^2^ Department of Psychiatry and Brain Center University Medical Center Utrecht Utrecht The Netherlands; ^3^ Department of Pediatrics Wilhelmina Children's Hospital Utrecht The Netherlands; ^4^ Dutch Autism & ADHD Research Center, Department of psychology University of Amsterdam Amsterdam The Netherlands; ^5^ Department of Psychiatry Radboud University Medical Center Nijmegen The Netherlands; ^6^ Child and Adolescent Psychiatry, Karakter Nijmegen The Netherlands; ^7^ Program in Genetics and Genome Biology, Research Institute The Hospital for Sick Children Toronto ON Canada; ^8^ Department of Psychiatry University of Toronto Toronto ON Canada; ^9^ Department of Psychology Wilhelmina Children's Hospital Utrecht The Netherlands

**Keywords:** 22q11DS, dimensions, neurodevelopment, psychiatry, symptom expression

## Abstract

**Background:**

The 22q11.2 deletion syndrome (22q11DS) is associated with a variety of neuropsychiatric outcomes that vary across deletion carriers. We adopted a dimensional approach to provide a comprehensive overview of neuropsychiatric symptom expression in adolescents with 22q11DS and further our understanding of the observed phenotypical heterogeneity.

**Methods:**

Participants were 208 adolescents with 22q11DS between 10 and 19 years old. Semi‐structured clinical interviews and IQ tests were used to quantify symptom expression on multiple symptom dimensions, some reflecting DSM‐IV diagnostic domains. We investigated symptom expression in those with and without a formal DSM‐IV classification and examined between and within symptom dimensions. We used correlation analyses to explore associations between different symptom dimensions.

**Results:**

We demonstrated inter‐individual differences in symptom expression, both between and within neuropsychiatric symptom dimensions. On most symptom dimensions, more than 50% of adolescents expressed at least one clinically relevant symptom. In addition, a significant proportion of youth without a formal DSM‐IV diagnosis reported clinically relevant symptoms (e.g. >85% of those without an ADHD diagnosis reported ADHD symptoms). The exploratory correlation analysis indicated mostly positive correlations between symptom dimensions.

**Conclusions:**

The finding that most adolescents with 22q11DS express neuropsychiatric symptoms, even in the absence of a DSM‐IV classification, has substantial ramifications for guiding adequate support. Findings may spur further research into the dimensional structure of neuropsychiatric symptoms in 22q11DS and aid in uncovering mechanisms that contribute to symptom expression. Ultimately, this provides leads to improve clinical care for 22q11DS and to understand phenotypical variation in other high‐risk genetic variants.

## Introduction

The 22q11.2 deletion syndrome (22q11DS; OMIM #188400, #192430) is a genetic disorder, caused by a hemizygous microdeletion of 0.7–3 million base pairs on chromosome 22. Among a rapidly growing list of genetic variants associated with high risk for the expression of neurodevelopmental or psychiatric disorders, 22q11DS is relatively common, with an estimated prevalence of 1 in 2000–4000 live births (Blagojevic *et al*. 2021). Additionally, 22q11DS is genetically well‐described, and increasingly, the genetic diagnosis of 22q11DS is made early in life. Together, this makes 22q11DS a promising model to understand potential mechanisms underpinning various neuropsychiatric conditions, but also to explain phenotypical variability, both in other rare genetic variants and in the general population (Insel 2010; Zinkstok *et al*. 2019; Fiksinski, Hoftman, Vorstman, & Bearden, 2023).

Despite sharing the same genetic risk factor, individual presentations of 22q11DS are characterised by a high degree of heterogeneity of clinical manifestations, including a range of physical symptoms, such as congenital cardiac and palatal abnormalities (McDonald‐McGinn *et al*. 2015), none of which is present in every individual with 22q11DS. The same clinical heterogeneity is observed for the expression of neurodevelopmental and psychiatric symptoms, hereafter referred to as neuropsychiatric symptoms, that comprise varying degrees of intellectual impairment and a range of psychiatric disorders (Schneider *et al*. 2014; Fiksinski *et al*. 2018). Despite recent progress (e.g., Davies *et al*. 2020), our understanding of factors contributing to the diversity and variable penetrance of neuropsychiatric symptoms in 22q11DS remains limited. This poses a significant challenge for clinical practice, in particular regarding the management of individual expectations and planning of (early) treatment strategies (Fiksinski *et al*. 2021). A better understanding of the underlying mechanisms driving neuropsychiatric symptom expression in 22q11DS is needed to further both research and clinical practice. To achieve this objective, obtaining a more fine‐grained phenotypic description of neuropsychiatric symptoms in 22q11DS is essential (Michelini, Palumbo, DeYoung, Latzman, & Kotov 2021; Jacquemont *et al*. 2022).

In the current literature, the neuropsychiatric symptoms associated with 22q11DS have been predominantly described from a categorical perspective (e.g. Schneider *et al*. 2014). This approach relies on diagnostic categories, each representing a separate psychiatric disorder, to classify patterns of neuropsychiatric symptoms (Kraemer 2007; Potuzak, Ravichandran, Lewandowski, Ongür, & Cohen 2012; Borsboom *et al*. 2016). Studies reporting the prevalence rates of diagnostic categories in 22q11DS indicate that autism spectrum disorder (ASD), attention deficit hyperactivity disorder (ADHD) and anxiety disorders are relatively prevalent in childhood, each affecting around 30% of the children with 22q11DS. Mood and psychosis spectrum disorders increase over the course of adolescence; around 25% of the individuals with 22q11DS will have a psychotic disorder in (early) adulthood (Schneider *et al*. 2014; Fiksinski *et al*. 2018; Jhawar *et al*. 2021). The prevalence rates of conduct behaviour and substance abuse are relatively low in individuals with 22q11DS (Fiksinski *et al*. 2018; Vingerhoets *et al*. 2019). Taken together, these categorical descriptions indicate that the 22q11.2 deletion is associated with a heterogeneous presentation of neuropsychiatric disorders, increasing the risk for some, but not all, carriers of this variant.

Even though categorical descriptions shed light on inter‐individual variability, they cannot fully capture the variation in neuropsychiatric symptom expression that may exist among individuals with 22q11DS. Firstly, a categorical approach classifies individuals in binary categories (i.e. an individual does or does not have a neuropsychiatric disorder), whereas symptoms of many diagnostic categories are continuously distributed in the general population (Lilienfeld & Treadway 2016; Haslam, McGrath, Viechtbauer, & Kuppens 2020). In addition, a categorical approach does not capture possible variation in the degree of expression of different core symptoms within each diagnostic category (Borsboom *et al*. 2016; Lilienfeld & Treadway 2016). Consequently, an exclusively categorical approach falls short in describing the type and severity of symptoms that are most frequently expressed in 22q11DS and may overlook potentially relevant symptoms in those individuals without a clinical diagnosis (Baker & Vorstman 2012).

To complement the categorical approach, a dimensional approach is required to describe neuropsychiatric symptoms in the general population (Sanislow *et al*. 2010; Michelini *et al*. 2021), as well as in carriers of rare genetic risk variants such as 22q11DS (Hiroi *et al*. 2013; Niarchou *et al*. 2017; Fiksinski *et al*. 2021). Here, neuropsychiatric symptoms are considered quantitative traits, or symptom dimensions, on which individuals vary in terms of severity (Kraemer 2007). Most commonly, the range of intellectual impairment is described both categorically and dimensionally in 22q11DS. The former approach has demonstrated that, on average 45% of the adults with 22q11DS has an intellectual disability (as indicated by intelligence quotient [IQ] < 70). The latter approach demonstrated that IQ scores in 22q11DS are normally distributed around a mean of 70 points (Swillen, Moss, & Duijff 2018). As this example demonstrates, a dimensional approach allows to provide a more detailed description of the 22q11DS‐associated symptom profile (Baker & Vorstman 2012; Niarchou, Martin, Thapar, Owen, & van den Bree 2015; Niarchou *et al*. 2017; Chawner *et al*. 2021). To further our insight into the neuropsychiatric phenotype associated with 22q11DS, we aim to provide an overview of neuropsychiatric symptoms in 22q11DS from a dimensional perspective, including multiple major neuropsychiatric domains.

## Methods

### Participants

A total of 208 adolescents with 22q11DS who visited the outpatient clinic at the psychiatry department of the University Medical Centre Utrecht (UMCU) in the Netherlands between 2002 and 2018 were included in the study. As part of the routine clinical care (Bassett *et al*. 2011), all patients with 22q11DS were offered to take part in a developmental and psychiatric assessment, regardless of having immediate developmental or psychiatric concerns. Inclusion criteria for participation in the study were (1) a genetically confirmed 22q11.2 deletion; (2) absence of an acquired brain trauma unrelated to 22q11DS; and (3) age at enrolment between 10 and 19 years old. Participants and, where relevant, their parents or legal guardians, provided written informed consent. The study was conducted in accordance with the Declaration of Helsinki (World Medical Association 2013) and was approved by the Medical Ethical review board of the UMCU.

#### Instruments

As part of the standard clinical assessment, three semi‐structured interviews were administered to all patients' parents or legal guardians to evaluate the presence of neuropsychiatric symptoms. The mood and psychosis sections of the Kiddie‐Schedule for Affective Disorders and Schizophrenia were used to measure symptoms associated with mood disorders and psychotic disorders (Kiddie‐SADS; Ambrosini 2000; Endicott & Spitzer 1978). A semi‐structured assessment of DSM‐IV Symptoms was used to assess the presence of symptoms associated with ADHD, anxiety disorder, disruptive behaviour disorders and eating disorders. A trained clinician rated for each symptom if it was absent (score 0), doubtfully present (score 1), mildly/moderately present (score 2) or strongly present (score 3). The Autism Diagnostic Interview‐Revised was used to measure symptoms associated with ASD (ADI‐R; Lord, Rutter, & Le Couteur 1994). During the same assessment, the patients' level of intellectual functioning was assessed with an age‐appropriate version of the standardised Wechsler scales of intelligence (Weschler 1997, 2008, 2014; Weschler & Naglieri 2006; Table S1). These tests provide scores for Full Scale IQ (FSIQ), Verbal IQ (VIQ) and Performance IQ (PIQ), all normally distributed with a mean = 100 and SD = 15 in the general population. All instruments were administered and/or scored by trained clinicians, and diagnostic classifications were made in accordance with DSM‐IV‐TR criteria, based on all available information (American Psychiatric Association 2000).

#### Symptom dimensions and standardised scores

We aimed to study the inter‐individual differences in the severity of symptom expression, beyond a categorical distinction (i.e. having or not having a clinical diagnosis). As such, our approach was to define different symptom dimensions that correspond to different neuropsychiatric domains, and subsequently, to quantify the expression of symptoms for each individual on each symptom dimension. Based on the structure of the clinical interviews and the intelligence assessments, we were able to define eight major symptom dimensions, as well as multiple minor symptom dimensions within each major symptom dimension (see Table 1 for a complete overview of the major and minor symptom dimensions and the instruments used for their operationalisation).

**Table 1:** Overview comprising the eight major symptom dimensions (**bold**) and minor symptom dimensions (*italics*), as well as the measures that were used to quantify participants' severity score for each symptom dimension, including the total number of items for each measure and the number of items needed to meet to fall in the clinical range (>CRC)

Symptom dimension	Instrument	*N* items Total	*N* items clinical range
**Intellectual functioning**	IQ test: Full‐Scale IQ score	—	IQ < 70
*Verbal Intelligence*	Verbal IQ score	—	IQ < 70
*Performance intelligence*	Performance IQ score	—	IQ < 70
**Attention deficit and hyperactivity**	DSM‐IV interview ‐ Main section: ADHD	18	12
*Attention deficit*	Subsection: Inattention	9	6
*Hyperactivity and impulsivity*	Subsection: Hyperactivity and Impulsivity	9	6
**Autism spectrum**	ADI‐R:	37	21
*Social interaction problems*	Subsection: Social interaction	16	10
*Communication problems*	Subsection: Communication	13	8
*RRBI*	Subsection: RRBI	8	3
**Mood**	KSADS—Main section: mood	30	8
*Depressive behaviour*	Subsection: Depression	24	5
*Manic behaviour*	Subsection: Mania	6	3
**Anxiety**	DSM‐IV interview ‐ Main section: Anxiety	25	13
*Generalised anxiety*	Subsection: Generalised Anxiety	8	5
*Separation anxiety*	Subsection: Separation Anxiety	8	3
*Obsessive compulsive behaviour*	Subsection: Obsessive Compulsive Disorder	9	5
**Psychosis spectrum**	KSADS—Main section: Psychosis	50	3
*Positive psychotic symptoms*	Subsection: Hallucinations and delusions	41	1
*Other psychotic symptoms*	Subsection: Other psychotic symptoms	9	2
**Disruptive behaviour**	DSM‐IV interview ‐ Main section: Behavioural disorders	24	7
*Oppositional defiant behaviour*	Subsection: Oppositional defiant disorder	8	4
*Conduct behaviour*	Subsection: Conduct disorder	16	3
**Eating behaviour**	DSM interview ‐ Main section: Eating disorders	16	11
*Anorexic behaviour*	Subsection: Anorexia	4	4
*Bulimic behaviour*	Subsection: Bulimia	6	6
*Other eating problems*	Subsection: Other	6	1

ADHD, attention deficit hyperactivity disorder; ADI‐R, Autism Diagnostic Interview–Revised; IQ, intelligence quotient; KSADS, Kiddie–SADS; RRBI, repetitive restricted behaviours and stereotyped Interests.

For the dimensions measured with any of the clinical interviews, we quantified symptom expression by computing the sum score of the items that belonged to each major symptom dimension and each minor symptom dimension. We used the outcomes of the intelligence measures (IQ scores) as quantification of the expression of symptoms on the dimension intellectual functioning. Specifically, we used the FSIQ scores for the major symptom dimension and the Verbal and Performance IQ scores for the minor symptom dimensions. To allow for comparison of the distributions of expressed symptoms across the different symptom dimensions, we standardised all sum‐ and IQ scores by computing percentage scores. For sum scores derived from the clinical interviews, we used the formula [(participant sum score on symptom dimension/the maximum possible sum score on that symptom dimension) × 100]. To transform the IQ‐scores to percentage scores, we first transformed all IQ scores >100 as a score of 100, and all scores <55 as a score of 55, so that the range of possible IQ scores only covered the level of ‘severely impaired’ to ‘average’ intellectual functioning. Then, we converted all IQ scores into percentage scores using the formula: [100 − ((IQ score −55)/45) × 100]. We inverted this percentage score to align it with the symptom distributions, such that a lower IQ score corresponds to more severity, that is, a higher percentage score. Hence, all symptom dimensions ranged from low percentage scores (i.e. few symptoms/problems) to high percentage scores (i.e. many symptoms/problems). In this study, we refer to these percentage scores as standardised scores.

#### Cut‐off scores and severity ranges

To support the interpretation of the distribution of scores within each symptom dimension, we defined two cut‐off values that enable us to divide each symptom dimension into three severity ranges. Here, it is important to note that these ranges function as a proxy for severity of symptom expression, and should therefore not be considered as equivalent to a categorical diagnosis.

First, the ‘Symptom Cut‐off score (SC)’ corresponds to the standardised score that represents the expression of one symptom on a given dimension. For the symptom dimensions that were measured with the clinical interviews, a symptom was considered present if an item was rated with a minimum score of 2. The SC was computed with the formula [(2/total number of items in that dimension) × 100]. For the symptom dimensions reflecting intellectual functioning, the SC corresponds with the standardised score that reflects an IQ score of 85 (i.e. >−1SD). We further refer to the range of standardised scores below the SC as the ‘normal range’.

Additionally, we computed for each major and minor symptom dimension a ‘Clinical Range Cut‐off score’ (CRC), but different approaches were taken to compute this score. Some of our major dimensions or minor dimensions directly mirror DSM‐IV categories. In these instances, the CRC corresponds to the standardised score that reflects the minimum number of symptoms that is required to meet the DSM‐IV diagnostic criteria using the formula [((minimum number of symptoms required for DSM‐IV diagnosis × 2)/total number of items in corresponding symptom dimension) × 100]. To calculate the CRC for those symptom dimensions that did not directly resemble a DSM‐IV diagnostic category, we used a slightly different procedure. For the minor dimensions that reflect a core symptom domain within a diagnostic category (e.g. ‘attention‐deficit’ within ‘ADHD’), we adopted the cut‐off criteria provided by the DSM‐IV if these were provided. For the major dimensions that by themselves did not mirror a DSM‐IV diagnostic category (e.g. ‘disruptive disorder’ that comprises oppositional defiant disorder and conduct disorder), we defined the CRC as sum of the items that is needed for a DSM‐IV diagnosis for their corresponding minor symptom dimensions (Table 1). For the symptom dimensions reflecting intellectual functioning the CRC corresponds to the standardised score that reflects an IQ score of 70 (i.e. >−2SD).

The range of standardised scores in between the SC and the CRC is referred to as the ‘subthreshold range’ and the range of scores above the CRC as the ‘clinical range’.

#### Analyses

Data manipulation, visualisation and analysis were done in RStudio version 4.0.2 (RStudio Team 2020). First, we calculated and visualised the distribution of standardised scores on both the major and minor symptom dimensions. In addition, we calculated the proportion of individuals within the normal, subthreshold and clinical range respectively for all of these symptom dimensions. To shed light on the range of expressed symptoms among individuals without a diagnosis, we described the distribution of standardised scores on each symptom dimension that corresponds to a clinical DSM‐IV diagnosis for the individuals without a clinical diagnosis. Finally, we used correlation analyses to explore the interrelationships between the different minor symptom dimensions. We correct for multiple comparisons with the Benjamini‐Hochberg procedure that controls the probability of false positives among all significant correlations, as is particularly recommended for conducting correlation analysis with an exploratory nature (Groenwold, Goeman, Cessie, & Dekkers 2021).

## Results

Dimensional overview of neuropsychiatric symptom expression in 22q11DS.

**Figure 1:**
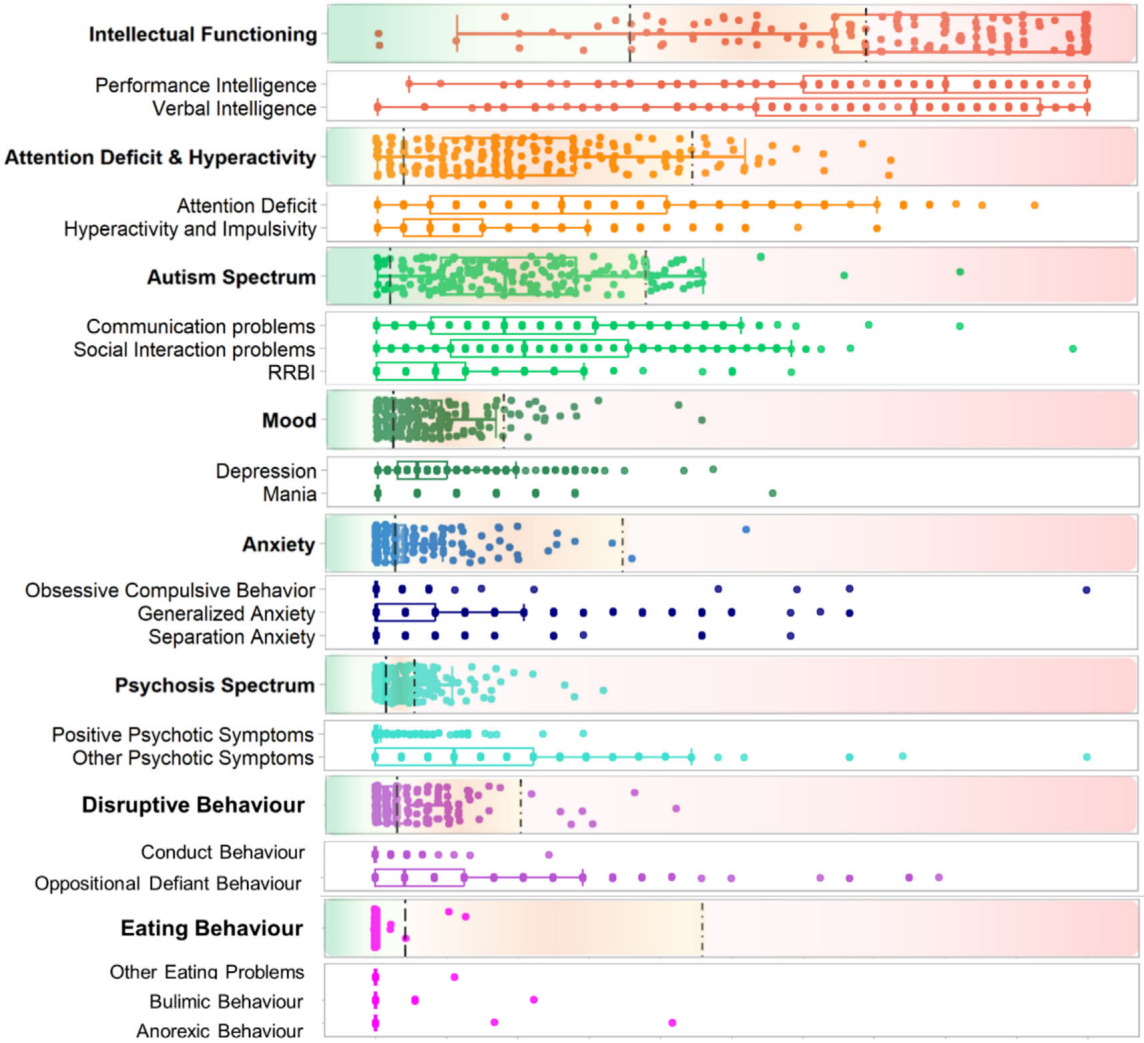
Boxplot presenting the standardised scores (%) on the major symptom dimensions (**bold**) and minor symptom dimensions for adolescents with 22q11DS. Individual dots represent individual participants. Participants with the same score share overlapping dots. RRBI, repetitive restricted behaviours and stereotyped interests. Note: Area to the left of the striped line = normal range; area between striped and dotted line = subthreshold range; area to the right of the dotted line = clinical range

Participants were on average 13.6 years old (SD = 1.90), and the total sample of 208 adolescents included 123 girls (59%) and 85 boys (41%). Figure 1 displays the distribution of the standardised scores of the adolescents with 22q11DS, within each of the eight major symptom dimensions and their corresponding minor symptom dimensions (see also Table S2).

### Major symptom dimensions

The range of standardised scores varied between the different major symptom dimensions, with especially the dimensions ‘Intellectual Functioning’, ‘Attention Deficit & Hyperactivity’ and ‘Autism Spectrum’ having a relatively large proportion of adolescents with high standardised scores, reflecting higher levels of symptom expression, as compared to other major symptom dimensions. Furthermore, standardised scores of the adolescents with 22q11DS varied within most major symptom dimensions, ranging from low to high levels of symptom expression. The data revealed that most adolescents had a standardised score that fell in the subclinical range on the majority of major symptom dimensions. Exceptions are the major dimension Intellectual Functioning, in which most scores fell in the clinical range, and the major dimension Eating Behaviour, in which most scores fell in the normal range. Of note, only 4% of scores fell in the normal range for the major dimension Autism Spectrum, indicating that 97% of the adolescents expressed at least one symptom on this dimension. In the major dimension Psychosis Spectrum, a relatively large proportion of the adolescents had a score in the clinical range, whereas, on average, standardised scores were relatively low on this dimension. Of note here is that the presence of one symptom was enough to exceed the cut‐off for the clinical range for the minor symptom dimension positive symptoms.

#### Minor symptom dimensions

Similar to observations of the major symptom dimensions, standardised scores of the adolescents with 22q11DS varied both between and within the minor symptom dimensions. The distribution of expressed symptoms varied between some minor symptom dimensions that belong to the same major symptom dimension. More specifically, within the major symptom dimensions Attention Deficit and Hyperactivity, Autism Spectrum, Mood, Anxiety and Disruptive Behaviour, we observed that the proportion scores that fell in the clinical range was much larger for one of the minor symptom dimensions. Exceptions were the two minor symptom dimensions within Intellectual Functioning, with a comparable proportion of adolescents with severity scores in the subclinical and clinical range. Additionally, the minor dimensions reflecting eating behaviour revealed little variation, and most of the adolescents had a standardised score of 0, indicating no symptom expression.

#### Dimensions in relation to categories

Table 2 provides an overview of the distribution of standardised scores on each major and minor symptom dimension, for the subset of adolescents with 22q11DS without a DSM‐IV diagnostic classification corresponding to this symptom dimension. This overview illustrates a degree of symptom expression that is comparable to the results for the total sample (Table S2). An exception seems the proportion of individuals with a standardised score exceeding the CRC on the minor symptom dimension ‘positive psychosis symptoms’. Here, it appears that individuals without a diagnostic classification of a psychotic disorder present with lower standardised scores (i.e. fewer symptoms), as compared to the total sample.

**Table 2:** Overview of the distribution of standardised scores on each major symptom dimension (**bold**) and minor symptom dimension (*italics*), only including the standardised scores of adolescents with 22q11DS who do not have a corresponding DSM‐IV diagnostic classification.

Symptom domains	DSM‐IV^§^	%^¶^	Median	IQR	%normal	%subthreshold	%clinical
**Attention deficit and hyperactivity**	ADHD	92.5	**16.7**	**20.8**	**11.4**	**77.7**	**10.9**
*Inattention*		—	25.9	29.6	19.0	57.1	23.9
*Hyperactivity and impulsivity*		—	11.1	18.5	31.7	61.2	7.10
**Autism spectrum**	ASD	55.6	**16.2**	**19.9**	**6.42**	**77.1**	**16.5**
*Social interactions problems*	—	—	18.8	27.0	9.17	69.7	21.1
*Communication problems*	—	—	18.0	23.0	17.4	67.0	15.6
*RRBI*	—	—	4.17	12.5	50.9	35.2	13.9
**Mood** †	—	92.9	5.56	6.94	18.5	71.2	10.3
*Depressive behaviour*	MDD	92.9	**6.94**	**8.33**	**20.7**	**59.2**	**20.11**
*Manic behaviour*	—	92.9	0	0	90.8	8.70	0.54
**Anxiety** †	—	92.4	**2.67**	**6.67**	**48.1**	**50.8**	**1.10**
*Generalised anxiety*	GAD	99.5	0	16.7	57.9	35.5	6.60
*Separation anxiety*	SAD	99.5	0	0	87.2	9.18	3.58
*Obsessive compulsive behaviour*	OCD	97.8	0	0	95.3	3.12	1.55
**Psychosis spectrum**	PD	86.7	**2.67**	**4**	**28.5**	**47.7**	**28.8**
*Other psychotic symptoms*	—	—	7.41	18.5	35.9	23.5	40.6
*Positive psychotic symptoms*	—	—	0	0.81	77.2	19.9	2.92
**Disruptive behaviour**	—	99	**1.45**	**7.25**	**53.0**	**43.4**	**3.54**
*Oppositional defiant behaviour*	ODD	99	4.17	16.7	54.5	34.3	11.1
*Conduct behaviour*	CD	100	0	0	94.5	4.50	1.00
**Eating behaviour**	—	100	**0**	**0**	**98.5**	**1.52**	**0**
*Anorexic behaviour*	AN	100	0	0	99.0	1.01	0
*Bulimic behaviour*	BN	100	0	0	99.5	0.51	0
*Other eating problems*	—	100	0	0	99.5	0	0.51

^†^ For the major dimensions Mood, Anxiety, Disruptive Behaviour and Eating Behaviour, only scores of participants without a DSM‐IV diagnosis in any of the corresponding diagnostic classifications were used.

^‡^ There was no registration of participants with a DSM‐IV diagnosis of bipolar disorder. We used scores of participants without a DSM‐IV diagnosis of MDD.

^§^ This column indicates the DSM‐IV diagnostic classifications that directly corresponds with a major or minor symptom domain.

^¶^ Indicating the proportion of individuals without a diagnostic classification, out of the total individuals with a score (see Table 2). Based on this information, the total number of individuals with a diagnostic classification can be computed (Example ADHD: 100% ‐ 92.5% = 7.5% of the sample had a diagnosis of ADHD).

^††^ As stated elsewhere, the prevalence rates of different neuropsychiatric conditions in 22q11DS vary across countries and cohorts. It has been suggested that this variation may occur due to various factors including differences in ascertainment referral or assessment procedures (Schneider *et al*. 2014; Fiksinski *et al*. 2018).

ADHD, attention deficit hyperactivity disorder; AN, anorexia nervosa; ASD, autism spectrum disorder, BN. bulimia nervosa; CD, conduct disorder; Clinical, clinical range; GAD, generalised anxiety disorder; MDD, major depressive disorder; normal, normal range; OCD, obsessive compulsive disorder; ODD, oppositional defiant disorder; PD, psychotic disorder; RRBI, repetitive restricted behaviours and stereotyped interests; SAD, separation anxiety disorder; Subthreshold, subthreshold range.

#### Correlations

**Figure 2:**
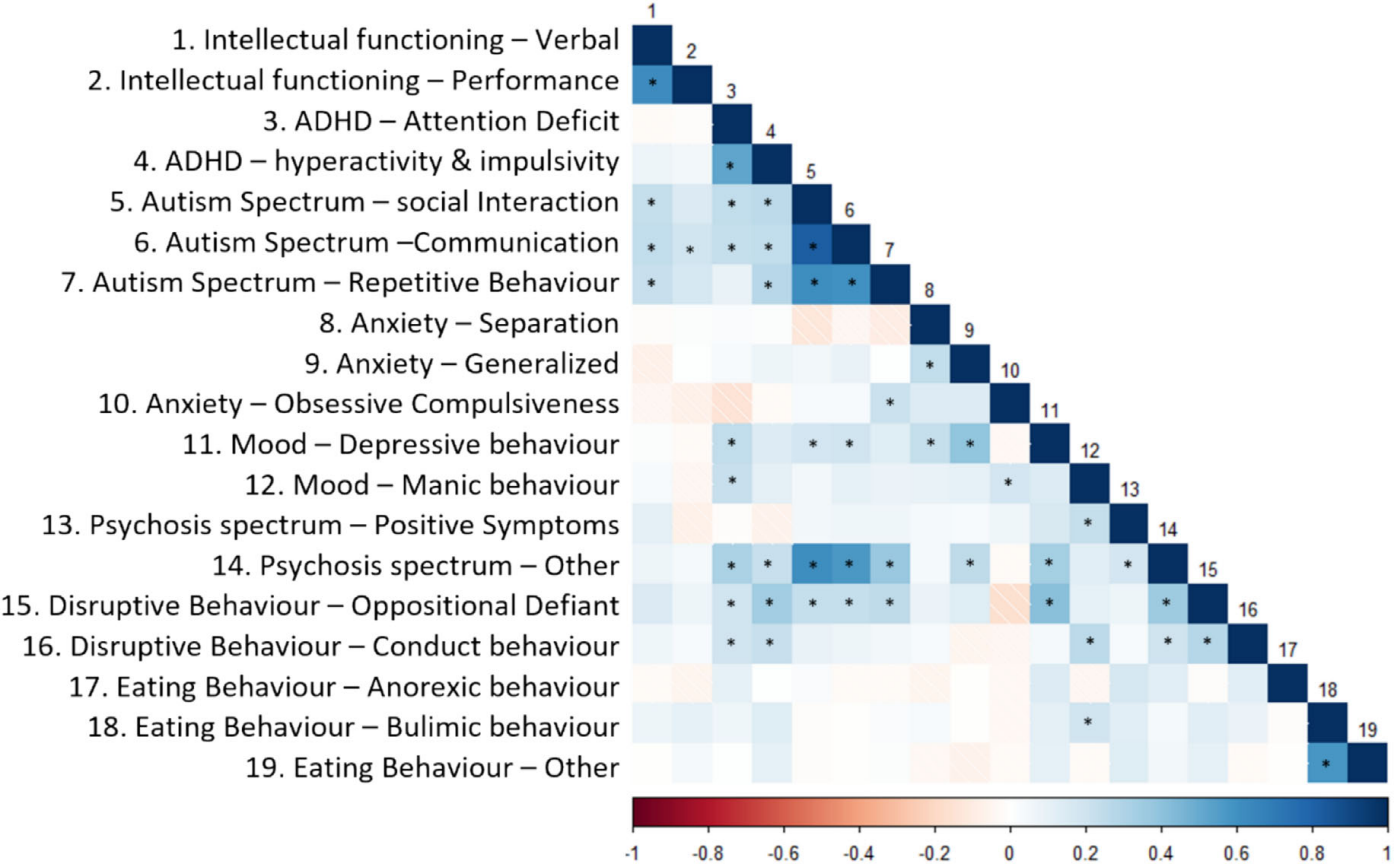
Results of Spearman correlation analyses, ranging from −1 (dark red) to +1 (dark blue), to explore the associations between scores on the minor symptom domains (*p < .05 after correction for multiple comparisons).

Given the skewed distribution of our data, we used Spearman correlation analyses to investigate the associations between all minor symptom domains (see Figure 2 and Table S3). Results mostly showed positive associations between minor symptom domains, indicating that higher levels of symptom expression on one minor symptom dimension were associated with higher levels of symptom expression on other minor symptom dimensions. We did not only observe such significant associations between minor symptom dimensions that belong to the same major symptom dimension (e.g. communication with social interaction in the Autism Spectrum dimension) but also significant cross‐dimension associations (e.g. anxiety‐generalised with mood depression). Furthermore, we observed that some minor symptom dimensions were associated with multiple other minor symptom dimensions (e.g. disruptive behaviour‐oppositional behaviour).

## Discussion

We used a dimensional approach to provide a comprehensive overview of neuropsychiatric symptoms in adolescents with 22q11DS. To this end, we quantified symptom expression within eight major symptom dimensions and multiple minor symptom dimensions. We found that individuals with 22q11DS experience a wide range of neuropsychiatric symptoms, which often do not meet the criteria for a full clinical diagnosis of a neuropsychiatric disorder.

Results of the present study indicate that adolescents with 22q11DS express more symptoms on the major symptom dimensions ‘Intellectual functioning’, ‘Attention deficits and hyperactivity’ and ‘Autism spectrum’ as compared to other major dimensions (e.g. ‘Eating behaviour’), which is in keeping with the previously reported prevalence rates of the different categorical diagnostic classifications in adolescents with 22q11DS (Schneider *et al*. 2014). Previous descriptions indicated that none of the neuropsychiatric disorders was completely penetrant in 22q11DS (Fiksinski *et al*. 2021). Our present overview takes a dimensional perspective and complements those previous descriptions by demonstrating a broad range of inter‐individual variation in the severity of neuropsychiatric symptom expression in most major and minor symptom dimensions. In line with previous observations (Niarchou *et al*. 2017), the results of the present study indicate that neuropsychiatric symptom expression in 22q11DS, similar to the general population (Haslam *et al*. 2020), is distributed continuously, rather than discretely.

We demonstrate that a dimensional approach allows to describe the degree of expression of different core symptoms within each neuropsychiatric category. For instance, we observed that the major symptom dimension attention deficit hyperactivity is mainly driven by the expression of symptoms on the minor dimension attention deficits in adolescents with 22q11DS, in line with earlier work (Niarchou *et al*. 2015). In addition, results of the current study indicate that symptoms associated with ASD seem more prominent in the domain of social communication and interaction and less clearly in the domain of restricted and repetitive behaviours in adolescents with 22q11DS. This is interesting, as this difference was not observed in young children with 22q11DS (Serur *et al*. 2019; Selten *et al*. 2023). However, in the present study, the number of questions probing the repetitive domain was substantially lower than for the other two domains, which may have influenced our results. Alternatively, it may be, similar to the general population, that repetitive behaviours in 22q11DS tend to decrease between early childhood to adolescence (Masjedi, Clarke, & Lord 2024). Overall, our finding align with descriptions, that autistic features are characteristic for individuals with 22q11DS, also in the absence of autism (Kates *et al*. 2007; Serur *et al*. 2019; Selten *et al*. 2023).

As has been demonstrated previously, individuals with 22q11DS present with subthreshold symptoms in multiple diagnostic categories, but most prominently involving psychosis (Weisman *et al*. 2017; Schneider *et al*. 2019), ASD (Serur *et al*. 2019; Chawner *et al*. 2021), ADHD (Klaassen *et al*. 2013; Niarchou *et al*. 2015) and anxiety (Klaassen *et al*. 2013). Our findings broaden this observation by explicitly demonstrating that those adolescents with 22q11DS without a formal psychiatric diagnosis still display a wide range of symptoms, often across several symptom neuropsychiatric domains, with, in some instances, scores in the clinical range.

To further characterise the 22q11DS symptom profile, we explored the associations between different minor symptom dimensions. Our data revealed multiple significant associations between various minor symptom dimensions, including between those minor symptom dimensions that belong to a different major symptom dimension (e.g. between minor dimensions within attention deficit and hyperactivity and autism spectrum). Moreover, it appeared that in some instances, a single minor symptom dimension was associated with multiple minor dimensions that belonged to a variety of symptom dimensions (e.g. mood depression). It could be that some of these observed correlations are (partly) due to overlapping symptoms between different minor symptom dimensions. A prime example of such a minor symptom dimension is other psychotic symptoms, containing symptoms associated with autism spectrum (e.g. difficulty connecting with others) and depression (e.g. flattened affect). Nonetheless, the highly correlated symptom domains in 22q11DS resemble observations in the general population that individuals with clinically relevant symptoms that belong to a particular diagnostic category are likely to present with increased rates of symptoms in other diagnostic categories (Lilienfeld & Treadway 2016).

### Strengths, limitations and future directions

A strength of the present study is that we measured the expression of neuropsychiatric symptoms in multiple major and minor symptom dimensions in a large sample of adolescents with 22q11DS, which allowed us to give a comprehensive overview of neuropsychiatric symptom expression in this population.

In this study, we used the sum of item scores within each symptom dimension as a means to indicate the severity of neuropsychiatric symptom expression for each of our participants. As a consequence, the symptom profile of individuals with the same sum score may differ. For instance, an individual having three items with the score 1, received the same sum score as an individual having one item with of the score 3. In addition, we chose the expression of one symptom as threshold for the subthreshold range, because we aimed to gain insight in the full range of inter‐individual variation of individuals below the clinical range. A consequence of this rather lenient cut‐off value is that our subthreshold range may not resemble the similar range in standardised instruments (e.g. those using a T‐score distribution). Furthermore, our subthreshold range allowed to demonstrate that a vast proportion of adolescents with 22q11DS have at least one symptom interfering with daily life functioning. However, our data do not demonstrate to what extent this proportion differs from the general population. Finally, the cut‐off for the clinical range for most of our symptom dimensions does not directly correspond to DSM criteria, which may limit their interpretability in relation to clinical reality. Therefore, further research employing different quantification strategies and including a typically developing control group might confirm our current results and improve our insight into the profile of symptom expression of adolescents with 22q11DS.Furthermore, it could be argued that results of this study are not representative for all individuals with 22q11DS, given that we used data that was collected at a psychiatry outpatient clinic. However, following the current international guidelines for care for individuals with 22q11DS (Bassett *et al*. 2011), all adolescents with 22q11DS were referred to this clinic regardless of having immediate psychiatric concerns, which may reduce recruitment bias. To confirm our suggestion that the study of 22q11DS could provide relevant insight into the inter‐individual variability in neuropsychiatric symptom expression in the general population, or other populations with rare genomic diseases, future studies are needed to compare the dimensional symptom profile of 22q11DS to that of other genetic populations.

In line with one previous study (Shankman *et al*. 2018), we showed that outcomes of clinical interviews can be used to quantify the expression of neuropsychiatric symptoms. This may be relevant for initiatives aiming to combine samples and looking for clinically relevant neuropsychiatric outcomes (e.g. Jacquemont *et al*. 2022). Moreover, this approach directly relates to clinical practice, as the dimensional approach allows to complement a categorical diagnosis with a measure of severity, in line with suggestions of the Diagnostic and Statistical Manual of Mental Disorders, 5th edition (DSM‐5; American Psychiatric Association 2013; LeBeau, Bögels, Möller, & Craske 2015). Likewise, future descriptions of neuropsychiatric outcomes in 22q11DS are recommended to include measures of functional outcomes, such as adaptive functioning or quality of life.

### Clinical and theoretical implications

Results of this study have some direct clinical implications. First, our approach highlights that there is a group of individuals with 22q11DS, who do not meet criteria for a formal diagnosis of a neurodevelopmental or psychiatric disorder, but do experience a wide range of neuropsychiatric symptoms. Even in the absence of such a clinical diagnosis, the presence of subthreshold symptoms may be associated with significant distress and impairment, particularly when these symptoms are expressed across several neuropsychiatric domains (Baker & Vorstman 2012). Therefore, findings of the present study strongly support the clinical guidelines for 22q11DS (Oskarsdóttir, Vujic, & Fasth 2004; Boot *et al*. 2023). These recommend a broad and repeated evaluation of both the level of cognitive functioning and presence of neuropsychiatric symptoms, for all children with 22q11DS, throughout development into early adulthood. Such repeated and thorough assessment might limit the risk of diagnostic overshadowing, which refers to overlooking neuropsychiatric symptoms due to the presence of another neuropsychiatric disorder or misattributing symptoms to another (e.g. social problems as a result of an intellectual disability; Fiksinski *et al*. 2021). A side effect of such diagnostic overshadowing is that some neuropsychiatric symptoms may not receive adequate clinical attention. On an individual level, a dimensional approach, complementary to diagnostic categories, may therefore help reveal an individual profile of vulnerabilities on a symptom level, which may have important implications for tailoring individual support. For instance, in addition to a formal diagnosis of ‘intellectual disability and ADHD’, a complementary dimensional approach would describe that this child may predominantly present with present with symptoms of inattention, generalised anxiety and disruptive behaviour.

Our findings fit a general tendency in neuropsychiatric research to complement traditional diagnostic categories with dimensional observations when studying factors that contribute to the development of psychopathology (e.g. Research Domain Criteria; Cuthbert 2015). To further identify the risk factors that contribute to inter‐individual variation of neuropsychiatric symptom expression in individuals with 22q11DS, it may be of interest to explore the use of a person‐centred approach: a data‐driven approach that can be used to cluster individuals based on similarities in their individual patterns of neuropsychiatric symptoms (Howard & Hoffman 2018; Djelantik, Robinaugh, Kleber, Smid, & Boelen 2020). Identification of such (transdiagnostic) symptom clusters in 22q11DS could thus pave the way for further studies to detect biological or environmental risk factors for these clusters. Ultimately, understanding such risk factors for neuropsychiatric symptom expression in 22q11DS may facilitate identification of neurobiological pathways of common mental disorders and may guide research into novel targets for therapeutic intervention (Zinkstok *et al*. 2019; Fiksinski *et al*. 2023).

## Conclusion

In this study, we used a dimensional approach to explore the expression of neuropsychiatric symptoms of adolescents with 22q11DS, complementary to a description in terms of diagnostic categories. This provides a fine‐grained description of the inter‐individual variation of neuropsychiatric symptom expression in this population. Our results are consistent with a body of research reporting on large phenotypic heterogeneity in individuals with 22q11DS and demonstrate a wide range of neuropsychiatric symptom expression among those adolescents without a formal DSM‐IV diagnostic classification. These findings may enhance our ability to manage clinical outcomes and to tailor clinical support and intervention for adolescents with 22q11DS. In addition, the presented dimensional overview may spur hypotheses for future studies aiming to investigate the biological and environmental mechanisms contributing to symptom expression in this genetic high‐risk model for common mental disorders.

## Source of funding

This research was supported by a grant from the Dutch Organization for Scientific Research (NWO; grant number 360‐89‐080). The funder was not involved in the study design, collection, analysis and interpretation of data, writing the report and in the decision to submit the article for publication.

## Conflict of interest

Jacob Vorstman has served as a consultant for NoBias Therapeutics Inc and received speaker fees from Henry Stewart Talks Ltd (both unrelated to the content of this manuscript). All other authors declare that there are no competing interests in relation to the subject of this study.

## Supporting information


**Table S1.** Overview of the instruments to measure the level of intellectual functioning (IQ tests), together with the proportion of individuals that completed each test.
**Table S2.** Medians and interquartile ranges (IQR) of standardised scores of the adolescents with 22q11DS on each major symptom dimension (**bold**) and minor symptom dimension (*italics*), as well as the proportions of adolescents with a standardised scores in the different severity ranges.
**Table S3.** Results of Spearman correlation analyses, to explore the associations between scores on the minor symptom domains (*BH *p* < .05).

## Data Availability

The data that support the findings of this study are available on request from the corresponding author. The data are not publicly available due to privacy or ethical restrictions.
