# Supplementary material for: A comprehensive overview of neuropsychiatric symptoms in adolescents with 22q11.2 deletion syndrome
Source: J Intellect Disabil Res. 2024 Oct 22;69(2):113–26. doi: 10.1111/jir.13196 (PMC11735868; doi:10.1111/jir.13196)
Supplement: Supplementary file 1 — Table S1. Overview of the instruments to measure the level of intellectual functioning (IQ tests), together with the proportion of individuals that completed each test. Table S2. Medians and interquartile ranges (IQR) of standardised scores of the adolescents with 22q11DS on each major symptom dimension (bold) and minor symptom dimension (italics), as well as the proportions of adolescents with a standardised scores in the different severity ranges. Table S3. Results of Spearman correlation analyses, to explore the associations between scores on the minor symptom domains (*BH p < .05). [file JIR-69-113-s001.docx]

**Supplementary Information**

**Appendix S1**

**Table S1**. Overview of the instruments to measure the level of intellectual functioning (IQ tests), together with the proportion of individuals that completed each test.

| **Name instrument** | **n** | **%** |
| --- | --- | --- |
| **WAIS-III** | 8 | 4,49 |
| **WAIS-IV** | 3 | 1,69 |
| **WISC-III** | 158 | 88,76 |
| **WISC-V** | 7 | 3,93 |
| **WNV** | 2 | 1,12 |

Abbreviations. WAIS III = Wechsler Adult Intelligence Scale – 3rd edition. WAIS IV = Wechsler Adult Intelligence Scale – 4th edition. WISC-III = Wechsler Intelligence Scale for Children – 3^rd^ edition. WISC-V = Wechsler Intelligence Scale for Children – 5th edition. WNV = Wechsler NonVerbal intelligence scale.

Note. The WNV does not make a differentiation between verbal IQ and performance IQ. For these two participants only the full scale IQ score was used.

**Table S2.** Medians and interquartile ranges (IQR) of standardised scores of the adolescents with 22q11DS on each major symptom dimension (**bold**) and minor symptom dimension (*italics*), as well as the proportions of adolescents with a standardised scores in the different severity ranges.

| Symptom domains | N | Median | | IQR | % normal range | | % subthreshold range | | % clinical range |
| --- | --- | --- | --- | --- | --- | --- | --- | --- | --- |
| Intellectual Functioning | **178** | **84.4** | **35.6** | | | **3.50** | | **23.0** | **45.5** |
| *Performance Intelligence* | 175 | 80.0 | 40.0 | | | 16.6 | | 28.6 | 54.9 |
| *Verbal Intelligence* | 174 | 75.6 | 40.0 | | | 10.3 | | 28.7 | 60.9 |
| Attention deficit & hyperactivity | **199** | **18.5** | **22.2** | | | **11.1** | | **77.9** | **11.1** |
| *Attention deficit* | 199 | 25.9 | 31.5 | | | 19.1 | | 56.8 | 24.1 |
| *Hyperactivity and impulsivity* | 198 | 11.1 | 18.5 | | | 31.8 | | 60.6 | 7.58 |
| Autism spectrum | **196** | **18.0** | **19.4** | | | **3.57** | | **79.8** | **16.3** |
| *Social interaction problems* | 196 | 20.8 | 25.5 | | | 9.18 | | 68.4 | 22.5 |
| *Communication problems* | 196 | 17.9 | 23.1 | | | 12.8 | | 73.5 | 13.8 |
| *RRBI* | 195 | 8.3 | 14.6 | | | 46.2 | | 40.0 | 13.9 |
| Mood | **198** | **5.6** | **7.8** | | | **18.9** | | **71.0** | **10.1** |
| *Depressive behaviour* | 198 | 6.9 | 8.3 | | | 20.7 | | 58.7 | 20.6 |
| *Manic behaviour* | 198 | 0 | 0 | | | 90.4 | | 9.09 | 0.51 |
| Anxiety | **198** | **2** | **6.7** | | | **50.0** | | **49.0** | **1.01** |
| *Generalised anxiety* | 198 | 0 | 16.7 | | | 58.1 | | 35.4 | 6.57 |
| *Separation anxiety* | 197 | 0 | 0 | | | 87.3 | | 9.14 | 3.55 |
| *Obsessive compulsive behaviour* | 197 | 0 | 0 | | | 94.9 | | 2.03 | 3.05 |
| Psychosis spectrum | **198** | **3** | **5.2** | | | **25.3** | | **42.4** | **32.3** |
| *Other psychotic symptoms* | 196 | 11.1 | 22.2 | | | 33.7 | | 20.9 | 45.4 |
| *Positive psychotic symptoms* | 197 | 0 | 1.63 | | | 68.5 | | 20.81 | 10.7 |
| Disruptive behaviour | **200** | **4.2** | **7.2** | | | **52.5** | | **44.0** | **3.50** |
| *Oppositional defiant*  *behaviour* | 200 | 1.4 | 0 | | | 54.5 | | 34.5 | 11.0 |
| *Conduct behaviour* | 200 | 0 | 16.7 | | | 94.5 | | 4.50 | 1.00 |
| Eating behaviour | **198** | **0** | **0** | | | **98.5** | | **1.52** | **0.00** |
| *Anorexic behaviour* | 198 | 0 | 0 | | | 99.0 | | 1.01 | 0.00 |
| *Bulimic behaviour* | 198 | 0 | 0 | | | 99.5 | | 0.51 | 0.00 |
| *Other eating problems* | 197 | 0 | 0 | | | 99.5 | | 0.00 | 0.51 |

Abbreviations. RRBI = repetitive restricted behaviours and stereotyped interests

Note. Numbers of complete data vary due to practical reasons, including lack of time during the assessment, non-compliance of a participant, and not being able to obtain information from external sources (e.g., school).

**Table S3.** Results of Spearman correlation analyses, to explore the associations between scores on the minor symptom domains (***BH p<.05**)

|  | IF_V | IF_P | ADH_A | ADH_H | AS_I | AS_C | AS_R | Anx_S | Anx_G | Anx_O | Mood_D | Mood_M | Psy_P | Psy_O | DB_O | DB_C | EB_A | EB_B | EB_N |
| --- | --- | --- | --- | --- | --- | --- | --- | --- | --- | --- | --- | --- | --- | --- | --- | --- | --- | --- | --- |
| IF_V | 1 |  |  |  |  |  |  |  |  |  |  |  |  |  |  |  |  |  |  |
| IF_P | **0.87*** | 1 |  |  |  |  |  |  |  |  |  |  |  |  |  |  |  |  |  |
| ADH_A | -0.24 | -0.22 | 1 |  |  |  |  |  |  |  |  |  |  |  |  |  |  |  |  |
| ADH_H | -0.09 | -0.09 | **0.73*** | 1 |  |  |  |  |  |  |  |  |  |  |  |  |  |  |  |
| AS_I | **0.26*** | 0.17* | **0.28*** | **0.31*** | 1 |  |  |  |  |  |  |  |  |  |  |  |  |  |  |
| AS_C | **0.28*** | **0.2*** | **0.25*** | **0.28*** | **0.98**** | 1 |  |  |  |  |  |  |  |  |  |  |  |  |  |
| AS_R | **0.29*** | 0.2* | 0.08** | **0.27*** | **0.86**** | **0.85*** | 1 |  |  |  |  |  |  |  |  |  |  |  |  |
| Anx_S | -0.28 | -0.21 | -0.16 | -0.23 | -0.47 | -0.41 | -0.46 | 1 |  |  |  |  |  |  |  |  |  |  |  |
| Anx_G | -0.36 | -0.26 | -0.07 | -0.1 | -0.11 | -0.09 | -0.2 | **0.4*** | 1 |  |  |  |  |  |  |  |  |  |  |
| Anx_O | -0.23 | -0.24 | -0,43* | -0.32 | -0.13 | -0.12 | **0.09*** | 0.18* | 0.15* | 1 |  |  |  |  |  |  |  |  |  |
| Mood_D | -0.33 | -0.35 | **0.24*** | 0.08* | **0.06*** | **0.05*** | -0.07 | **0.25*** | **0.55*** | -0.26 | 1 |  |  |  |  |  |  |  |  |
| Mood_M | -0.25 | -0.34 | **0.17*** | -0.01 | -0.17 | -0.14 | -0.15 | 0.05 | 0.03 | **0.18*** | 0* | 1 |  |  |  |  |  |  |  |
| Psy_P | -0.09 | -0.3 | -0.25 | -0.4 | -0.18 | -0.18 | -0.18 | -0.04 | -0.07 | 0.02 | 0.07* | **0.22*** | 1 |  |  |  |  |  |  |
| Psy_O | -0.06 | -0.12 | **0.39*** | **0.28*** | **0.8**** | **0.78*** | **0.57*** | -0.27 | **0.16*** | -0.27 | **0.36*** | -0.08 | **-0.03*** | 1 |  |  |  |  |  |
| DB_O | 0.02* | -0.05 | **0.31*** | **0.46*** | **0.27*** | **0.26*** | **0.22*** | -0.07 | 0.09 | -0.51* | **0.51*** | -0.05 | -0.14 | **0.44*** | 1 |  |  |  |  |
| DB_C | -0.09 | -0.13 | **0.29*** | **0.27*** | -0.01 | -0.05 | -0.08 | -0.13 | -0.26 | -0.28 | -0.11 | **0.29*** | -0.13 | **0.11*** | **0.32*** | 1 |  |  |  |
| EB_A | -0.24 | -0.27 | 0.04 | -0.16 | -0.16 | -0.23 | -0.27 | -0.16 | -0.09 | -0.17 | 0.09* | -0.19 | 0.21* | -0.01 | -0.16 | 0.08 | 1 |  |  |
| EB_B | -0.1 | -0.03 | -0.11 | -0.02 | -0.35 | -0.35 | -0.3 | -0.09 | -0.24 | -0.22 | -0.09 | **0.09*** | 0.06 | -0.32 | -0.03 | -0.06 | -0.16 | 1 |  |
| EB_N | -0.14 | -0.07 | -0.17 | -0.05 | -0.27 | -0.28 | -0.23 | -0.17 | -0.27 | -0.19 | -0.07 | -0.18 | 0.11 | -0.23 | -0.02 | -0.19 | -0.11 | **0.82*** | 1 |

*p<.05, **p<.001 . Abr. IF_V = Intellectual Functioning – Verbal intelligence. IF_P = Intellectual Functioning – Performance intelligence. ADH_A = attention deficit hyperactivity – attention deficit. ADH_H = attention deficit hyperactivity – hyperactivity & impulsivity. AS_I = Autism Spectrum – Social Interaction problems. AS_C = Autism Spectrum –Communication problems. AS_R = Autism Spectrum – Restricted Repetitive Behaviours & Stereotyped Interests. AS-D = Autism Spectrum – Developmental Delay. Anx_S = Anxiety – Separation anxiety. Anx_G = Anxiety – Generalised anxiety. Anx_OC = Anxiety – Obssessive Compulsive behaviour. Mood_D = Mood – Depressive behaviour. Mood_M = Mood – Manic behaviour. Psy_P = Psychosis spectrum – Positive Symptoms. Psy_O = Psychosis spectrum – Other symptoms. DB_O = Disruptive Behaviour – Oppositional Defiant behaviour. DB_C = Disruptive Behaviour – Conduct behaviour. EB_A = Eating Behaviour – Anorexic behaviour. EB_B = Eating Behaviour – Bulimic behaviour. EB_N = Eating Behaviour – other eating problems
